# Combined use of *Chrysoperla rufilabris* (Neuroptera: Chrysopidae) and Cry3Aa for improved control of *Leptinotarsa decemlineata* (Coleoptera: Chrysomelidae)

**DOI:** 10.1093/jisesa/ieaf058

**Published:** 2025-07-29

**Authors:** Matheus Moreira Dantas Pinto, Swati Mishra, Sergio Antonio De Bortoli, Juan Luis Jurat-Fuentes

**Affiliations:** Department of Entomology and Plant Pathology, University of Tennessee, 370 E. J. Chapman Dr, Knoxville, TN 37996, USA; Department of Plant Protection, School of Agricultural and Veterinarian Sciences, São Paulo State University, Access Prof. Paulo Donato Castellane s/n, Jaboticabal, São Paulo 14884-900, Brazil; Department of Entomology and Plant Pathology, University of Tennessee, 370 E. J. Chapman Dr, Knoxville, TN 37996, USA; Department of Plant Protection, School of Agricultural and Veterinarian Sciences, São Paulo State University, Access Prof. Paulo Donato Castellane s/n, Jaboticabal, São Paulo 14884-900, Brazil; Department of Entomology and Plant Pathology, University of Tennessee, 370 E. J. Chapman Dr, Knoxville, TN 37996, USA

**Keywords:** predator, CPB, Bt, biological control, potato

## Abstract

The Colorado potato beetle (CPB, *Leptinotarsa decemlineata* (Say)) is the most important defoliator of solanaceous crops. Control of this pest is hindered by its ability to develop resistance to insecticides, including insecticidal proteins from *Bacillus thuringiensis* (Bt) Berliner. Therefore, it is important to find alternative tools that may be combined into an integrated pest management approach for CPB control. In this study, we evaluated the efficiency of the combined use of the Cry3Aa protein from Bt and *Chrysoperla rufilabris* (Burmeister) in controlling CPB. Control of CPB larval second instar at different densities by 3 larval instars of the predator was tested in potato leaves treated with an LC_50_ concentration of the Cry3Aa protein. Potato leaf damage was evaluated in experiments where CPB larvae were fed with leaves treated with Cry3Aa protein and exposed to predator larvae for 7 days or until all CPB larvae were dead. Results show that all 3 instars of the predator presented a type II functional response for all conditions evaluated. The first and second larval instars of *C. rufilabris* larvae attacked twice the number of prey (1.34–2.91 and 2.9–4.75, respectively) when CPB larvae were fed on leaves with the Cry3Aa protein. A complete reduction in the number of CPB larvae and lower levels of potato leaf damage was observed when the predator larvae were present. These results support the combined use of the Cry3Aa protein with *C. rufilabris* in increasing the efficiency of CPB control.

## Introduction

Potato (*Solanum tuberosum* L., Solanaceae) is considered one of the most important food crops globally (Devaux et al. 2020), representing an essential element for food security and a relevant source of income in developing countries (Zhang et al. 2017). Potato crops are grown in 158 countries worldwide, and total production increased from 376 Mt in 2022 to 383 Mt in 2023. However, in North America, Europe, and Asia, potato growers face severely reduced yields from Colorado potato beetle (CPB, *Leptinortarsa decemlineata* (Say), Coleoptera: Chrysomelidae) infestations (Weber 2003, Alyokhin et al. 2008, FAO 2023). Left unmanaged, both larvae and adults of CPB can cause extensive defoliation and damage to stems and exposed tubers (Alyokhin et al. 2022). Estimated yield losses from CPB damage are variable but can reach up to billions of dollars annually (Nault and Kennedy 1998, Skryabin 2010). High feeding rates combined with high fertility and adaptability to different habitats (Maharijaya and Vosman 2015, Cingel et al. 2016), make CPB a major concern for farmers, triggering insecticidal applications even when populations are below economic thresholds (Alyokhin et al. 2022).

Historically, CPB control has been mainly based on chemical control (Grafius and Douches 2008) and has resulted in the evolution of resistance to most of the products registered for CPB control (Alyokhin et al. 2008, Szendrei et al. 2012, Siddiqui et al. 2023). The primary and most common approach to preventing resistance is the rotation of insecticides with different modes of action during the crop season (Roush 1989), thereby reducing the selection pressure on the pest population (Chen et al. 2021). However, CPB resistance has been recorded for 56 active ingredients (Mota-Sanchez and Wise 2024), and there is evidence for the ability of CPB to evolve resistance to recently commercialized RNAi biopesticides (Mishra et al. 2021) without fitness costs (Pinto et al. 2023). This ability to evolve resistance greatly hinders integrated pest management (IPM) strategies (Siddiqui et al. 2023), which have been proposed as the only sustainable CPB control method (Alyokhin et al. 2015).

The evolution of pesticide resistance and the growing demand for environmentally friendly and organic production have led producers to increase the use of biological control tools and include them in IPM rotations (Gassmann et al. 2008, Sparks et al. 2020). Among biological control alternatives, the gram-positive entomopathogenic bacterium *Bacillus thuringiensis* (Bt) Berliner, is considered the most successful microbial pesticide (Raymond and Federici 2017). The effectiveness of Bt mostly relies on the production of insecticidal crystal (Cry) proteins (Jurat-Fuentes and Crickmore 2017) and the Cry3A family is well known for its toxicity against coleopteran hosts (van Frankenhuyzen 2009). Transgenic potato producing the Cry3Aa protein was commercialized for CPB control in 1995 but was withdrawn from the market in 2001 due to consumer resistance and anti-biotech pressure (Thornton 2003). However, biopesticides based on Bt bacteria-producing Cry3Aa remain used for CPB control (Wraight and Ramos 2017).

Traditional biological control strategies for CPB control include the use of insect predators such as lacewings, which usually feed on soft-bodied arthropods like aphids, caterpillars, whiteflies, and mites (Legaspi et al. 1994, Giles et al. 2000, Hernández-Juárez et al. 2016, Wang et al. 2023). The green lacewing *Chrysoperla rufilabris* (Burmeister) (Neuroptera: Chrysopidae) occurs naturally in various agroecosystems, yet it performs better in areas with higher levels of humidity like potato fields (Daane 2001). This predator is easy to rear in the laboratory and is available for commercialization in potato-producing countries, including the United States (Tauber et al. 2000). Like other natural enemies the use of lacewings is a prospective alternative for sustainable CPB management (Nordlund et al. 1991, Sablon et al. 2013), however, they are not able to keep CPB densities below economically damaging levels by themselves (Hough-Goldstein et al. 1993). Our study aimed to evaluate the efficiency of the combined use of *C. rufilabris* and Cry3Aa for controlling CPB in potato plants.

## Material and Methods

### Insects

The GC (for General Colony) population of CPB used in this study was described before (Mishra et al. 2021). The colony was derived from eggs, larvae, and adults of CPB collected from 13 locations in 9 US states. The *C. rufilabris* colony used in this study originated from eggs obtained from a commercial insectary (Beneficial Insectary, Redding, CA, USA).

### Insect Rearing

Adults of the GC colony were maintained in aluminum screened cages (61 × 61 × 122 cm, W × H × L) kept in a greenhouse under controlled conditions (27 ± 3 °C, RH: > 70%, and 18L:6D). Eggs were collected every 2 days from the cages with adults and transferred to larval cages (same as described above) until pupation. Larvae and adults of CPB were fed ad libitum on potato plants (*Solanum tuberosum* var. Desiree) grown pesticide-free at the University of Tennessee AgResearch Plateau and Education Center (Crossville, TN, USA) in 11.3 L (3 gal) containers.

The colony of *C. rufilabris* was reared following the methodology adapted by de Freitas (2001). Twenty pairs of adult *C. rufilabris* were kept in an incubator (Percival, Perry, IA) under controlled conditions (26 ± 1 °C, RH: 75%, and 12L:12D) in cylindrical cages (16 × 20 cm, D × H) with the inside covered with paper as oviposition substrate. The cages were covered with cheesecloth on the top and a plastic pot (17 × 8 cm, D × H) on the bottom. The adult *C. rufilabris* diet was a mix of honey and brewer’s yeast (1:1 ratio) (de Freitas 2001) and was provided daily on a piece of moistened cotton attached to the top of the cage. Eggs were collected every 3 days using a stiletto blade and then placed in covered cups with 3 eggs per cup. During the entire immature phase, the predator was kept in the cups and fed daily ad libitum with eggs of *Ephestia kuehniella* (Zeller) (Lepidoptera: Pyralidae) obtained from a commercial insectary (Beneficial Insectary).

### Cry3Aa Protein

The *B. thuringiensis* subsp. *morrisoni* biovar *tenebrionis* strain was obtained from the *Bacillus* Genetic Stock Center (Columbus, OH, USA). An isolated colony was used to inoculate 2 L flasks containing sterile 0.5 L of 1/3 tryptic soy broth (TSB) medium. The cultures were incubated for 3 days at 28 °C with 160 rpm constant agitation. After confirming > 90% sporulation through microscopic observation, cultures were centrifuged for 10 min (10,000 rpm) at 4 °C, and the resulting pellets were resuspended in 1 M NaCl containing 0.1% Triton X-100. This centrifugation-resuspension washing cycle was repeated a total of 3 times, followed by 3 similar centrifugation-resuspension cycles using distilled water. The final pellet was resuspended in solubilizing solution (50 mM Na_2_CO_3_, 0.1% β-mercaptoethanol, 0.1 M NaCl) and incubated overnight at 30 °C and 200 rpm constant agitation. The solution was centrifuged for 30 min at 4 °C (14,500 rpm), and the supernatant was loaded on a HiTrap Q HP column pre-equilibrated with buffer A (50 mM Na_2_CO_3_, pH 9) connected to an AKTA-Pure FPLC system (GE Healthcare). Elution was performed using a linear gradient of buffer B (buffer A with 1 M NaCl). Fractions containing a band of the expected Cry3Aa protoxin size (~73 kDa) were identified by SDS-10%PAGE and then pooled and quantified with BSA as a standard (Bradford 1976), and kept at −80°C until used for experimentation.

### Functional Response

**Fig. 1.**
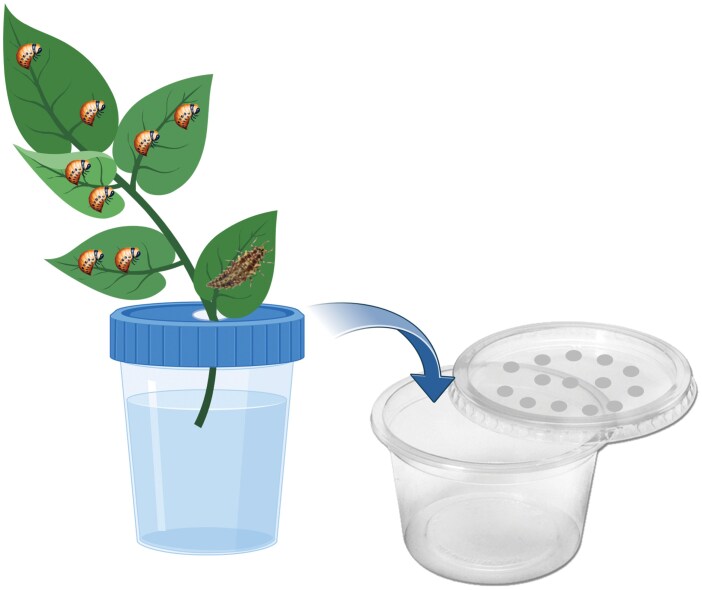
Schematic illustration of a test arena used in the bioassays. The figure was created with BioRender.com.

The functional response of *C. rufilabris* preying on CPB larval second instar was assessed under laboratory conditions. For all larval instars of the predator, prey densities were defined after a preliminary test to identify the minimum and maximum number of larvae consumed by the predator in 24 h. The densities were 2, 4, and 8 CPB larvae for the first; 2, 4, 8, and 16 for the second; and 2, 4, 8, 16, 32, 64, and 128 for the *C. rubfilabris* larval third instar. Fifteen replicates (one predator larva per replicate) were performed for each density. Each test arena consisted of a potato branch (~16 cm long) with 5 leaves each that were treated with the LC_50_ concentration of Cry3Aa against CPB (4.22 µg/ml), previously estimated in our laboratory for the GC strain (Mishra et al. 2021), or with distilled water and 0.1% Tween-20 as a control. To maintain turgidity, potato branches were placed in a plastic cup containing 50 ml of distilled water through a hole (0.4 cm diameter) on the cup lid surface. The branches were treated by dipping the leaves for 10 s in test or control solutions and then kept in a flow chamber for drying. CPB larvae were released on the branches with a paintbrush before adding the predator larvae. Test arenas were placed inside plastic containers (2 L), as illustrated in Fig. 1. Larval *C. rufilabris* (24-h-old) were starved for 12 h before experiments. Prey consumption was recorded after 24 h by counting the number of larval CPB completely or partially consumed.

### CPB Larval Survival and Damage to Potato Leaves

CPB larval survival and defoliation damage caused on potato leaves for each treatment were recorded for 7 days. The treatments included T1—Water + 0.1% Tween-20; T2—LC_50_ of Cry3Aa at 4.22 µg/ml + Tween 0.1%; T3—Water + third larval instar of *C. rufilabris*; and T4—LC_50_ Cry3Aa at 4.22 µg/ml + third instar larval of *C. rufilabris*. The potato branches were dipped and dried as described for functional response assays. Fifteen replicates (one predator larva per replicate) were performed. Ten CPB and one *C. rufilabris,* second and third larval instar, respectively, were released per arena. The third larval instar of *C. rufilabris* was used based on the best predation results obtained from functional response bioassays. To estimate the percentage of defoliation, potato branches were examined against a white background, and images were captured with a camera mounted on a stand at a constant distance (20 cm). The leaf area before and after consumption was measured using ImageJ software based on Richardson et al. (2001).

### Statistical Analysis

The functional responses exhibited by the predators were estimated from the shape of the functional response curve by performing logistic polynomial regression analyses using PROC CATMOD in SAS Software (SAS Institute Inc 2021). The relationship between the proportion of the original densities as a function of the number of CPB larvae consumed was fitted with the following polynomial function:


$$\begin{array}{l} \;\frac{\exp\;(P_{0}\;+\;P_{1}N_{0}\;+\;P_{2}N_{0\;}^{2}+\;P_{3}N_{0}^{3}}{1+(P_{0}\;+\;P_{1}N_{0}\;+\;P_{2}N_{0}^{2}\;+\;P_{3}N_{0}^{3}} \end{array}$$ (1)

where: $N_{a}$ represents the number of CPB larvae consumed; $N_{0}$ the number of CPB larvae offered; and *P*_0_, *P*_1_, *P*_2_, and *P*_3_ represent the intercept, constant linear, quadratic, and cubic coefficients, respectively. The sign of the linear coefficient was used to determine the shape of the functional response curve. A significantly negative linear coefficient (*P*_1_ < 0) and a positive linear coefficient (*P* > 0) indicate types II and III functional responses, respectively (Juliano 2001).

The attack rate ($a^{\prime }$, time to kill the prey) and the handling time (Th, the amount of time handling each prey) parameters were also generated from the functional response. Briefly, a non-linear regression of a ‘random predator equation’ as described by Rogers (1972), using PROC NLIN in SAS Software (SAS Institute Inc 2021) was performed as described by Juliano et al. (1993), and the parameters were compared based on the confidence intervals (95% CL) obtained, as follows:


$$\begin{array}{l} Na=\;N_{0}\left\{1-\exp\left[a^{\prime }\left(T_{h}NaT\right)\right]\right\} \end{array}$$ (2)

where: Na represents the number of prey attacked; $N_{0}$ the density of prey offered; $a^{\prime }$ the attack rate; $T_{h}$ the handling time; and T the time of predator exposure to the prey (24 h/$T_{h}$). The difference among treatments was considered statistically significant (α = 0.05) if the 95% CL overlapped between the treatment means (Juliano 2001, Dami et al. 2023). Moreover, the number of larval CPB survivors as a function of the initial number of larval CPB, as well as the defoliation percentage caused along the bioassay period (7 days) on Cry3Aa treated and untreated potato leaves were analyzed using the regression analysis performed by PROC REG in SAS Software (SAS Institute Inc 2021).

## Results

**Fig. 2.**
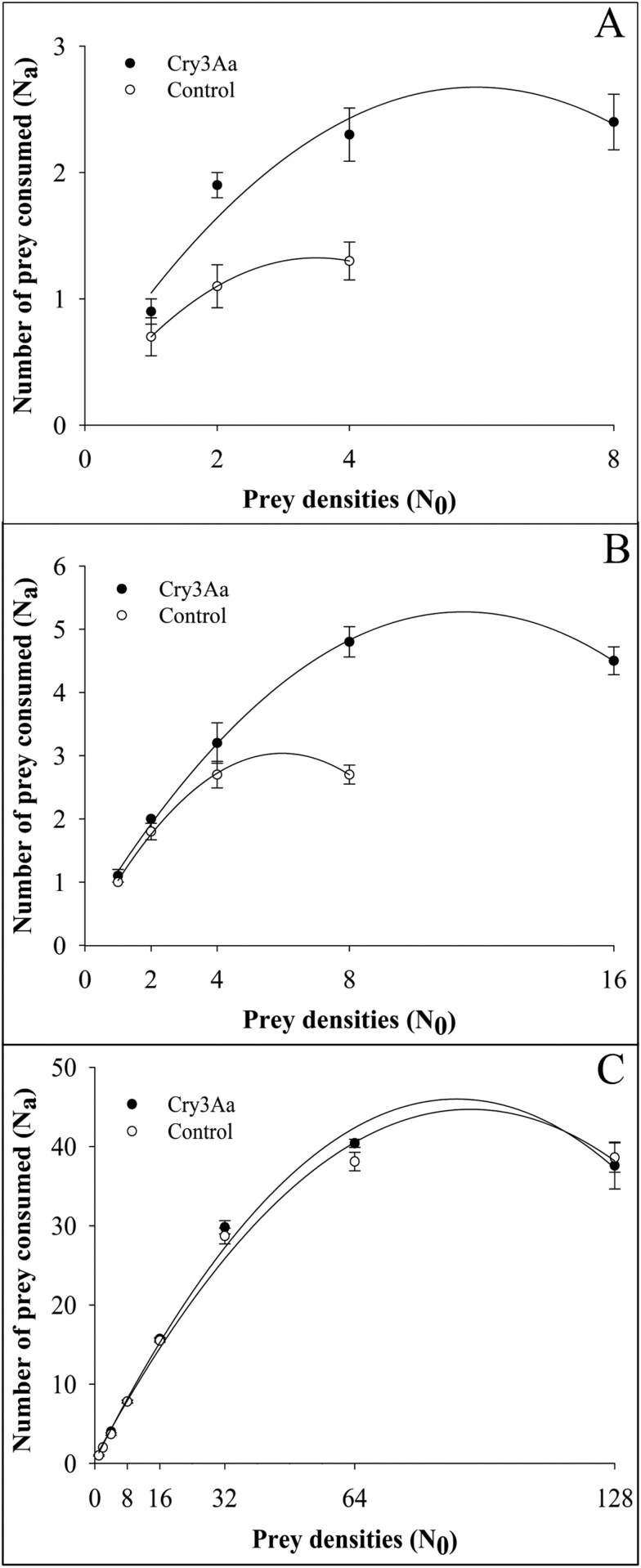
Functional responses of larval *Chrysoperla rufilabris*: A) first instar; B) second instar; C) and third instar, preying on larval second instar of *Leptinotarsa decemlineata* fed on Cry3Aa-treated (black circles) and control (open circles) potato leaves. Data shown represent means of larvae preyed ± SE from 15 replicates monitored after 24 h.

No differences were detected in the functional responses of the predator preying on larval CPB that fed on Cry3Aa-treated or untreated potato leaves. The logistic regression analysis showed significantly negative linear and positive quadratic coefficients for all 3 instars of the predator (Table 1), which indicates a type II functional response so that the number of prey consumed increases until the plateau of satiation (Fig. 2A–C). No differences in the attack rate ($a^{\prime }$) between treatments were found for the 3 instars of the predator (Table 2). However, there was a significant reduction in handling time ($T_{h}$) and about a 2-fold increase in the estimated number of prey attacked ($T/T_{h}$) (Table 2). The first and second larval instar of the predator doubled their attack capacity when released to prey upon the second larval instar of CPB fed on Cry3Aa-treated leaves (Table 2). No differences in the number of preyed larval CPB fed with control or Cry3Aa-treated leaves were observed for the third larval instar of the predator, which presented a handling time ($T_{h}$) between 0.57 and 0.60 hours and an estimated number of prey attacked between 40 and 42.

**Table 1.** Estimated parameters resulting from polynomial logistic regression analysis of the proportion of larval *Leptinotarsa decemlineata* on Cry3Aa treated and untreated potato leaves preyed upon by *Chrysoperla rufilabris* over a 24-h period

Instar	Treatments	Parameters	Values	df	χ^2^	*P*	Type
1^st^	Control	Intercept (*P*_0_)	2.5721	1	8.13	0.004	II
		Linear (*P*_1_)	−0.8041	1	9.74	0.001	
		Quadratic (*P*_2_)	–	–	–	–	
	Cry3Aa	Intercept (*P*_0_)	2.9125	1	28.04	<0.001	II
		Linear (*P*_1_)	−0.4485	1	30.09	<0.001	
		Quadratic (*P*_2_)	–	–	–	–	
2^nd^	Control	Intercept (*P*_0_)	2.9551	1	28.4	<0.001	II
		Linear (*P*_1_)	−0.4552	1	30.59	<0.001	
		Quadratic (*P*_2_)	–	–	–	–	
	Cry3Aa	Intercept (*P*_0_)	2.2780	1	49.98	<0.001	II
		Linear (*P*_1_)	−0.205	1	63.91	<0.001	
		Quadratic (*P*_2_)	–	–	–	–	
3^rd^	Control	Intercept (*P*_0_)	4.5516	1	161.28	<0.001	II
		Linear (*P*_1_)	−0.0882	1	87.0	<0.001	
		Quadratic (*P*_2_)	0.00036	1	43.29	<0.001	
	Cry3Aa	Intercept (*P*_0_)	5.4203	1	145.25	<0.001	II
		Linear (*P*_1_)	−0.1032	1	80.48	<0.001	
		Quadratic (*P*_2_)	0.000422	1	42.35	<0.001	

**Table 2.** Attack rate ($a^{\prime }\;$) expressed in h^−1^, handling time $\left(T_{h}\right)$, and estimated number of prey attacked with their corresponding 95% confidence limits (CL) during the observation period (*T* = 24 h/*T*_*h*_) for *Chrysoperla rufilabris* preying on larval *Leptinotarsa decemlineata* fed on Cry3Aa-treated and control potato leaves

Instar	Treatment	$a^{\prime }$	$T_{h}$	$T/T_{h}$
1^st^	Control	0.1a (0.03–0.23)	17.84a (13.02–22.65)	1.34a (1.05–1.84)
	Cry3Aa	0.14a (0.03–0.25)	8.23b (7.48–8.99)	2.91b (2.66–3.2)
2^nd^	Control	0.14a (0.03–0.24)	8.26a (7.51–9.07)	2.9a (2.64–3.19)
	Cry3Aa	0.07a (0.03–0.12)	5.05b (4.66–5.44)	4.75b (4.41–5.15)
3^rd^	Control	0.01a (0.005–0.01)	0.60a (0.56–0.64)	40.0a (37.5–42.8)
	Cry3Aa	0.0087a (0.007–0.01)	0.57a (0.54–0.59)	42.1a (40.6–44.4)

Different letters indicate significant difference among treatments based on non-overlapping 95% CL.

Within each line, there is no significant difference among treatments if the 95% CL overlaps.

**Fig. 3.**
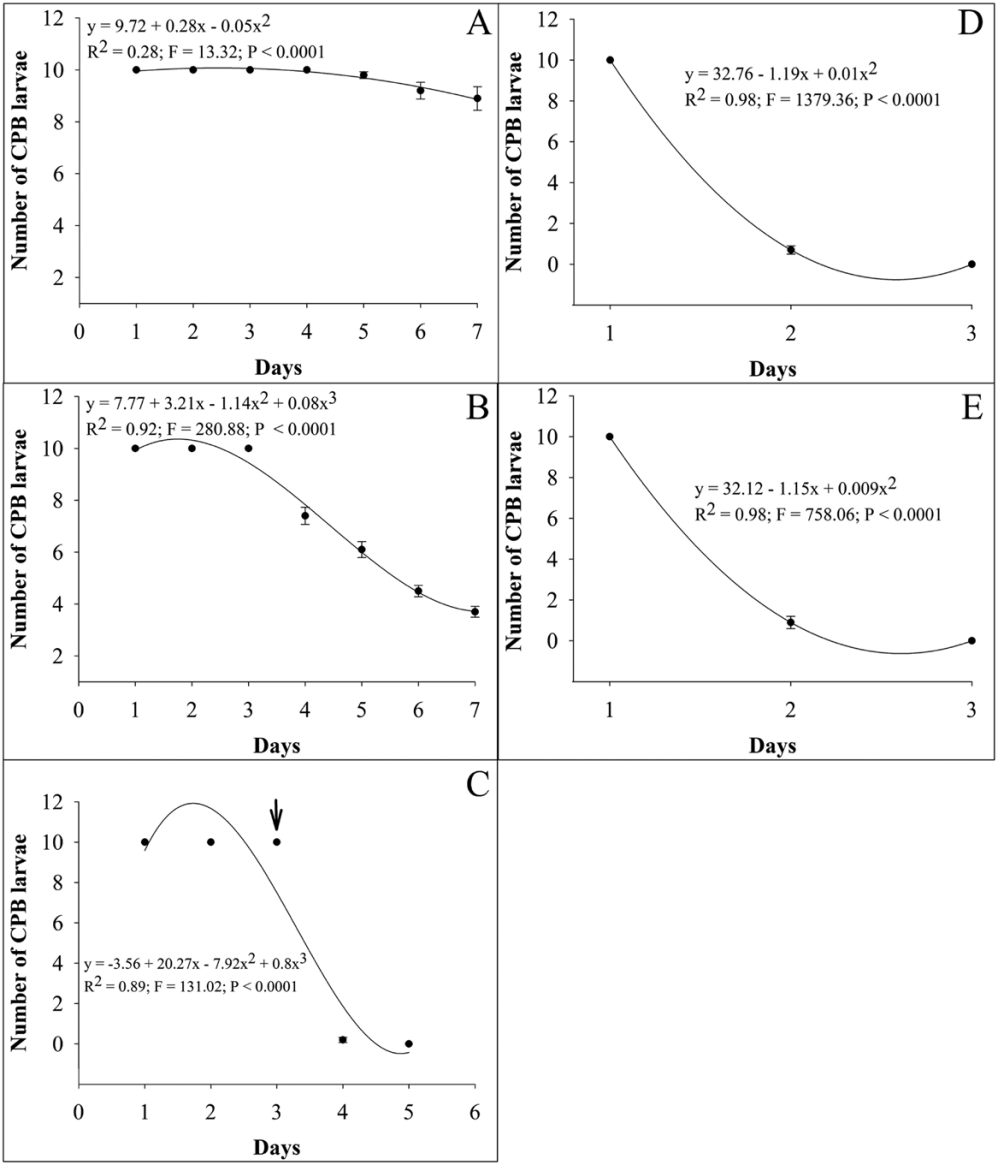
Mortality of larval second instar of *Leptinotarsa decemlineata* feeding on A) control potato leaves; B) Cry3Aa-treated potato leaves; C) Cry3Aa-treated potato leaves for 3 days followed by the release of a third larval instar of *Chrysoperla rufilabris* (arrow indicates the day of predator release); D) Cry3Aa-treated potato leaves for 3 h followed by release of a third larval instar of *Chrysoperla rufilabris* larva; and E) control potato leaves for 3 h followed by release of a third larval instar of *Chrysoperla rufilabris*. Data shown represent means of larvae ± SE from 15 replicates monitored every 24 h.

High survival of larval CPB was observed during the bioassay period when no method of control (biological or microbial control agent) was applied (Fig. 3A). Reduced survival was observed when larval CPB were fed on potato leaves treated with Cry3Aa for 7 days (Fig. 3B). When third larval instar of *C. rufilabris* was released after larval CPB had been feeding for 3 days on Cry3Aa-treated potato leaves, a drastic reduction in the number of surviving larval CPB was observed after 2 additional days (Fig. 3C). In all cases when larval *C. rufilabris* were used individually (Fig. 3D) or combined with exposure to Cry3Aa (Fig. 3E), all larval CPB were attacked and consumed in 2 days.

**Fig. 4.**
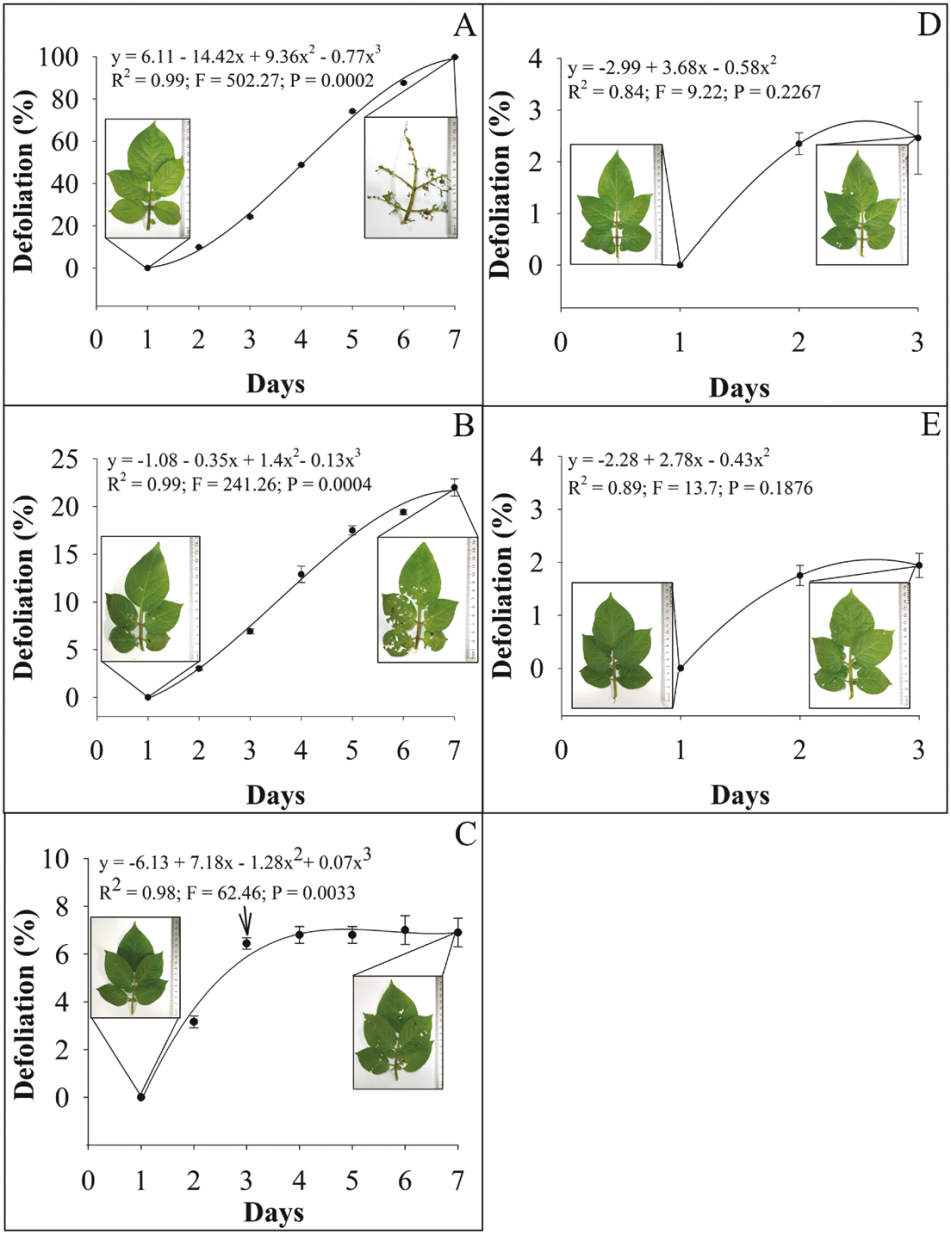
Defoliation caused by larval second instar of *Leptinotarsa decemlineata* on A) control potato leaves; B) Cry3Aa-treated potato leaves; C) Cry3Aa-treated potato leaves for 3 days followed by the release of a third larval instar of *Chrysoperla rufilabris* (arrow indicates the day of predator release); D) Cry3Aa-treated potato leaves for 3 h followed by the release of a third larval instar of *Chrysoperla rufilabris*; and E) control potato leaves for 3 h followed by release of a third larval instar of *Chrysoperla rufilabris*. Potato leaf pictures in each graph represent defoliation observed at the beginning (left) and at the end (right) of each bioassay. Data shown represent means of defoliation percentage ± SE from 15 replicates monitored every 24 h.

When no control tool was used against larval CPB, 100% defoliation was observed on potato leaves (Fig. 4A). When potato leaves treated with Cry3Aa protein were used, the level of defoliation was reduced to about 25% (Fig. 4B). There was a similar level of defoliation observed at the third day of the bioassay (Fig. 4B and C). However, the percentage of defoliation did not increase after releasing the third instar of *C. rufilabris* (Fig. 4C). Defoliation after the release of the third larval instar of *C. rufilabris* did not reach 3% independently of whether the potato leaves were treated with Cry3Aa or not (Fig. 4D and E).

## Discussion

Previous studies showed the lacewing predation potential on CPB (Nordlund et al. 1991, Hough-Goldstein et al. 1993, Sablon et al. 2013). Our study extended these observations to test predation when combined with a Cry3Aa Bt toxin treatment. Results support that the combined use of biological and microbial control approaches is beneficial to control CPB in potatoes. All 3 larval instars of *C. rufilabris* presented a type II functional response to increasing densities of the second larval instar of CPB offered in all treatments. This type II functional response is the most reported in multiple lacewing species preying on different pests (Hassanpour et al. 2011, Costa et al. 2020, de Oliveira Pimenta et al. 2020, Dami et al. 2023). Predators that present this type of functional response increase prey consumption as prey density increases until a plateau of satisfaction is reached (Holling 1959, 1961).

Feeding of the larval CPB on the potato leaves treated with Cry3Aa caused a positive influence on the predation ability of larval *C. rufilabris* in 2 ways, especially for first and second larval instar. First, it reduced handling time, defined as the time needed by the predator to find, capture, and feed on the prey. In turn, this increased the estimated number of prey attacked. These observations are in direct agreement with previous reports of alterations in handling time and an increase in *Ceraeochrysa cincta* (Schneider) consumption of *Plutella xylostella* (L.) eggs and larvae when exposed to a Bt-based insecticide (de Oliveira Pimenta et al. 2020). The increase in the number of prey consumed is favored by the commonly observed initial paralysis or slower movement after Cry intoxication (Jurat-Fuentes and Jackson 2012), thus making the insect more vulnerable to attack by predators (Genovart et al. 2010). In this scenario, debilitated prey can be advantageous for the performance of the predator, especially if the prey is larger than the predator. This may explain the increase in the estimated number of prey attacks for the first and second larval instar of *C. rufilabris*, which are smaller than the second larval instar of CPB. Another hypothesis that may explain the higher number of prey attacked when CPB fed on leaves treated with Cry3Aa is that sick prey may be nutritionally deficient compared to healthy prey, so a predator may need more sick prey to be satisfied (Salama et al. 1999, Lawo et al. 2010, Svobodová et al. 2017). However, this did not seem to influence the predatory behavior of the third larval instar of *C. rufilabris*, which presented a higher and similar estimated number of prey attacked in both conditions tested.

It is important to note that none of the control tactics tested herein provided complete protection (0% defoliation). This is expected from the experimental design since the Cry3A toxin concentration used (LC_50_) could have allowed some CPB feeding to continue (Salehian et al. 2021), and a larva of *C. rufilabris* would only feed on one larva of CPB at a time, allowing the others larval CPB to consume plant tissue (Supplementary Video S1). Thus, the pest:predator ratio greatly impacts the damage intensity (Zhang et al. 2022), with lower ratios resulting in less damage. The functional response data generated in this study provides insights for more effective strategies in the field. The higher and shorter attack rate (40–42.1) and handling time (0.57–0.60 h) observed for the third larval instar of *C. rufilabris* indicate this instar is preferred in cases of high incidence of the pest. On the other hand, the combination with Cry3Aa is strongly recommended for improving the performance of the first and second larval instar of *C. rufilabris* for CPB control.

Studies exploring interactions between control tools and lacewings are extremely important to assist in the selection of compounds compatible with lacewings to be used in IPM strategies. Several studies have reported incompatibility between chemical compounds used in IPM and lacewings (Cordeiro et al. 2010, Amarasekare and Shearer 2013, Rugno et al. 2019, Shan et al. 2020, Farias et al. 2023). In contrast, our study demonstrates a case of compatibility and synergic effect between biological and microbial control alternatives. This compatibility between lacewings and Bt toxins is supported by previous reports. For example, Cry1Ab toxin is harmless to larvae of *Chrysoperla carnea* (Stephens) (Romeis et al. 2004), Cry2Aa present in transgenic rice pollen and artificial diet is safe for adults of *Ceraeochrysa sinica* (Tjeder) (Wang et al. 2012), and trypsinized Cry1Fa and Vip3Aa have no detrimental effects on *Chrysopa pallens* (Rambur) offered through artificial diet (Ali et al. 2017). In general, Bt toxins are harmless for the development of lacewings (Ali et al. 2018).

For pest species with a history of insecticide resistance, such as CPB (Mota-Sanches and Wise 2024), the search for different modes of action is crucial, and the use of predators against CPB should be further explored. In the context of resistance management, the rotation of modes of action is key to delaying the development of resistance (Liu et al. 2014, Stenberg 2017, Nauen et al. 2019), especially since recurrent cases of resistance have reduced the effectiveness of control technologies in the field (Furlong and Zalucki 2010, Bahar et al. 2012, Liu et al. 2014, Nauen et al. 2019). The results herein demonstrate that the presence of the predator was crucial for the elimination of larval CPB that could survive when only one of the control methods was used. The commercial availability of *C. rufilabris* in some countries like the United States plays an important role in the decision for growers to include this predator in CPB IPM programs.

Historically, *C. rufilabris* is one of the first lacewing species to be used in augmentative releases for biological control (Daane 2001) due to the ability of this species to feed on various prey, including potato pests (Nordlund et al. 1991). Thus, it is possible to implement applied CPB control programs through augmentative releases of this species, which already occur naturally in agroecosystems. The implementation process for a biological control program can be tedious, yet the choice of an adapted, mass-reared, and commercially available predator could make the implementation more practical and less costly.

In conclusion, the present study presents experimental evidence supporting the combined use of 2 bioagents for improved control of CPB in the laboratory setting. Application of Cry3Aa or *C. rufilabris* individually provides CPB control and reduces plant damage levels, but their combined use introduces 2 distinct modes of action to reduce the risk of resistance while providing redundant killing. As suggested by Bahar et al. (2012) for *Mallada signatus* (Schneider) and Bt cotton against *Helicoverpa armigera* (Hübner), the predatory performance of *C. rufilabris* can serve as a second line of defense against any Cry3Aa survivors, potentially removing possible Cry3Aa-resistant CPB genotypes. Moreover, the use of Cry3Aa decreased the handling time and increased the estimated number of prey attacked by *C. rufilabris*. It is important to emphasize that the experiments tested here do not consider some factors that may influence predator performance in the field, such as intraspecific competition with other natural enemies, different stages of the pest, variation of abiotic parameters, and preference for other pests. Future studies should examine these interactions under semi-field and field conditions to better understand the influence of these factors on the performance of Cry3Aa and *C. rufilabris* for CPB control.

## Supplementary Material

ieaf058_suppl_Supplementary_Videos_S1
